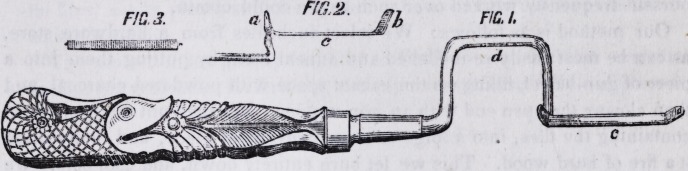# File Carriers

**Published:** 1847-03

**Authors:** 


*File Carriers.*—We have had, from time to time, many ingenious contrivances for holding files, and for doing away with the necessity of crooked files. But most of these have gone into disuse, partly from the fact that it was inconvenient to use in them ordinary files, but more from the fact that the files were held in place by moveable parts and screws, which rendered it almost impossible to keep them from rusting in the joints and about the screws. We have expended several dollars for file carriers, but have not used one for two or three years, for the above reasons.

To obviate the defects above alluded to, we have been led to construct one without moveable fixtures, which is represented in the following cut:

This instrument was very beautifully gotten up by Mr. Arnold, of Baltimore. After referring to the drawing it hardly needs description. It is made with a double bend to bring the file in a line with the handle, and, as there is nothing moveable, it of course requires a *pair.* The portion marked *c*, is a spring and through the arms *a* and *b*, there are square mortices, to receive the ends of the file and to keep it from turning. It will be perceived, by examining the cut, that the arm *b* does not come off at right angles, and the object of this shape must be apparent. The *file* is prepared by making each end square, corresponding with the size of the mortices in the arms, and is adjusted to the carrier by first putting one end of the file into the arm *a*, and pressing down the other end into the mortice *b*, the spring constituting that portion of the instrument between the arm, yields sufficient to admit of this. It will be readily perceived that the file may be easily taken out, while it could not well be thrown in an opposite direction; also, that the arm *a* sustains almost the entire strain made upon the instrument; the arm *b* serving to steady the file. We have here a file carrier entirely divested of joints, screws, or any fixture which may not be as readily kept clean, as any other instrument whatever, and the file being held by its two *ends,* admits of having one of any shape adapted to it.

Not only so, it admits of using the file in four different ways, which cannot be effected by any which we have hitherto seen.

For example, suppose we are using a half-round file, we may use the convex surface downward or upward, and so of either edge. This is certainly a matter of great convenience, both in separating between back teeth, or filing the tops of molar teeth, which have become frail, or the plugs after they are inserted. These files may be prepared from ordinary ones, by grinding the ends to fit the holes, or may be made to order, of the right form, by the manufacturer having the exact dimensions of the mortices in a given carrier.

If thin files are to be used in it, whose thickness is less than the diameter of the holes, a back may be fitted as directed in regard to the files, the sides of which are cleft, and the separating files fastened in this with shellac.

As many dentists are not located so as to have access to a file manufacturer, it may not be improper to add, in this connection, some directions for converting common files into such as are proper for the dentist's use. Not being able at all times to obtain files of a desirable shape, we have ourself frequently worked over such as we could obtain.

Our method is as follows: We select such files from a hardware store, as can be most easily re-modelled and anneal them by putting them into a piece of gun-barrel, filling up the vacant space with powdered charcoal, and then closing the open end with an iron stopper. We then put this gun-barrel containing the files, into a forge or some convenient place, and build upon it a fire of hard wood. This we let burn entirely down, and cool before we disturb it; after which time the files will be found to be as soft as steel is susceptible of being made, while they are hardly discolored by the heat. The reason of thus conducting the operation will be apparent to all who know that the scaling, consequent upon heating iron or steel, is produced by contact of air, or its oxygen. By this method we entirely exclude the air, and the files come out perfectly free from scales. They are now ready to be worked into any form or shape we may desire, either *by bending* them, which may be done cold, or, *by cutting or filing them* into any shape we may choose.

This accomplished, we next harden them, which must also be done without scaling them. To do this, we again put them into the gun-barrel, or any other iron box which can be made tight, and subject them to the strong heat of a forge. When heated nearly to whiteness, the box is to be suddenly plunged into a large vessel of *cold* water. The files are now ready for use. This is a very convenient and economical way of preparing files, and will often enable the operator to secure particular shapes, which he could not otherwise readily obtain. The same process of annealing and hardening will apply to any other instrument where it is desirable not to oxydise, or scale it.—*Syracuse Ed.*

## Figures and Tables

**Figure f1:**